# On the Support of Scientific Workflows over Pub/Sub Brokers

**DOI:** 10.3390/s130810954

**Published:** 2013-08-20

**Authors:** Augusto Morales, Tomas Robles, Ramon Alcarria, Edwin Cedeño

**Affiliations:** Department of Telematics Engineering, Technical University of Madrid, Av. Complutense 30, Ciudad Universitaria, 28040 Madrid, Spain; E-Mails: trobles@dit.upm.es (T.R.); ralcarria@dit.upm.es (R.A.); edwinc@dit.upm.es (E.C.)

**Keywords:** scientific workflow, publish/subscribe, distributed execution models, brokers, logic gates, workflow patterns

## Abstract

The execution of scientific workflows is gaining importance as more computing resources are available in the form of grid environments. The Publish/Subscribe paradigm offers well-proven solutions for sustaining distributed scenarios while maintaining the high level of task decoupling required by scientific workflows. In this paper, we propose a new model for supporting scientific workflows that improves the dissemination of control events. The proposed solution is based on the mapping of workflow tasks to the underlying Pub/Sub event layer, and the definition of interfaces and procedures for execution on brokers. In this paper we also analyze the strengths and weaknesses of current solutions that are based on existing message exchange models for scientific workflows. Finally, we explain how our model improves the information dissemination, event filtering, task decoupling and the monitoring of scientific workflows.

## 1. Introduction

A workflow management system (WfMS) is a piece of software that provides the infrastructure to setup, execute, and monitor workflows. These systems enable the “extraction” of process management from the application software, in order to achieve communication, system integration, process optimization and control. Nowadays, WfMS are very popular in business environments where workflows are well determined, ordered and tightly coupled with the computing resources that support them. WfMS are based on well-known business standards such as the Business Process Execution Language (BPEL) and Business Process Model and Notation (BPMN). These standards allow different entities to coordinate tasks by exchanging information in a simple and almost pervasive way—through web services.

A Scientific Workflow (SWf) is a special type of workflow that solves a complex scientific problem that is supported by a special WfMS called Scientific WfMS. As business workflows, SWfs are composed of several tasks that are coordinated by a global task scheduling system running in the SWfMS. The SWf's execution is divided into two layers. The *data plane* exchanges the execution information of an activity (e.g., a sensor output, the results of an image or weather analysis, or a cell-behavior). The *control plane* exchanges the activation or de-activation orders that allows the synchronization in the execution process of activities (e.g., to initialize a simulation). Thus, the control plane directly supports the global task scheduling as it decides where, when and how to execute tasks. Scientific workflows share some of the characteristics of business workflows [1] such as information filtering, process monitoring, and the necessity of a logical ordering of tasks that have to be carried out. Nevertheless, it has been proven that current business-oriented WfMS [2], and communication models barely support [3–5] the requirements of SWfs in terms of event dissemination, task decoupling, flexibility and scalability. SWfs are expected to be a more dynamic series of ordered tasks, changing inputs/outputs, and fluctuations of the logical relationships between participants. They also targets distributed environments with heterogeneous entities executing tasks with a high level of time, space and synchronization decoupling. As an example, initiatives such as DATAGRID [6], Open Science Grid [7], and XSEDE [8] provide systems and guidelines for executing SWfs and exploit the benefits of grid environments.

As communication over grid environments involves many challenges [9,10], one of the key issues in SWf research is the coordination of distributed workflows for a more efficient message exchange Thus, in order to improve this exchange it is necessary to take into account the runtime communication needs of a workflow, the logical relationships between its participants, and the type of tasks they execute. In addition, there are still challenges regarding how to improve the execution of SWfs by taking advantage of all the knowledge obtained from previous business-workflow research, and communication models that target loosely coupled systems. Hence, it has been proven that large-scale SWfs require models capable of providing an improved set of communication capabilities not only in parties that execute tasks, but also in entities bounding them. As an example, SWf platforms such as Taverna [11] and Pegasus [12], make use of grid infrastructures where workflow participants are heterogeneous in terms of location, processing power and network capabilities; however, little effort has been put into research on how the logical relationships between workflows' fragments influence the message exchange and the underlying protocols. Taverna also takes advantage of a Web Service Infrastructure [13] in order to improve its extensibility and compatibility with Web-based services.

Besides the multiple open-issues [9] in the SWfs area, we tackle the problem of supporting SWfs over grid-based environment in order to improve the event dissemination in the control layer. Therefore, we define key elements that enable the execution of SWf over the Publish/Subscribe (Pub/Sub) event layer. In our model we take as inputs two aspects: the message exchange in the control plane and the logical relationships between tasks. Even if the message exchanging of SWfs has been tackled with web-based technologies, it is has been proven that in highly distributed environments, using centralized solutions (at the event dissemination level) offers a low level of parallelism, communication decoupling and independence among participants.

The solution proposed in this paper exploits the logical relationships between fragments of a SWf and exposes abstract solutions, instead of directly tackling the message exchange aspect in the data plane with new protocols [14] or middlewares [15]. For this purpose, we use the Publish/Subscribe paradigm as the core communication model of our proposals. In addition, as one of the main requirements of SWf is the monitoring and failure recovery [16], we also define self-healing mechanisms for the proposed models in runtime, so our solutions maintain loosely coupled communications and fulfill the level of abstraction and dynamism required by SWf.

The structure of the paper is as follows: Section 2 describes the characteristics of scientific workflow models and presents the Pub/Sub-based model we use throughout the whole article. Section 3 presents the initialization process of a SWf, the broker reference architecture we have defined in order to improve the message exchange in the control plane, and procedures for recovering bindings in SWfs. In Section 4 we justify the qualitative advantages of our model by firstly grouping existing models, systems and implementations, into common categories based their communication solutions; and then analyzing their trade-offs in terms of event dissemination, workflow patterns and other communication needs in runtime. In Section 5 we analyze related works and finally in Section 6, we end with conclusions and suggestions for future works.

## 2. Scientific Workflow Modeling

Scientific workflows management systems (SWfMS) consist of several long-running data transformation steps while processing large amounts of data, coordinating and controlling the global workflow scheduling and monitoring underlying sub-tasks. In this process of decoupling the control and data planes from the task execution, the details of its invocation are hidden from the scientist. The high degree of dynamism inherent to these systems is not easily modeled or scaled [17] by a business WfMS, which provides orchestration with a centralized scheduling environments which also usually implement centralized messages in the control plane. On the other hand, a simple choreography approach cannot be used as it is difficult to keep track of all the task instances and workflow activities at any given time [18]. Hence, in order to support the previous characteristics, we use a Pub/Sub model for delivering these control messages in the whole SWf execution. Pub/Sub systems are composed of three main components: publishers, which are the content producers, subscribers, that express their willingness to consume specific content; and finally brokers, that put publishers and subscribers in contact by storing and forwarding information.

### 2.1. Overall SWf Scenario

SWfs share the same well-known workflow design patterns [19] as business workflows, as they are also composed by a set of logically connected tasks, therefore, as a continuation of previous works [20,21], we extended our workflow modeling, from the message exchange perspective, provide abstract model for supporting six workflow patterns over a Pub/Sub broker and present their advantages in comparison with other approaches in terms of information delivery and task decoupling.

In this paper, we consider a SWf scenario with complex interactions between tasks (e.g., change the running conditions, stop and re-initialize) over a grid scenario and following a direct acyclic graph execution. In these interactions, processing and communication resources must be dynamically shared between task instances as nodes are working at full capacity and can go off-line due to changes in the network topology. We also consider that parallel tasks may fail, so failure handling and compensation mechanisms are needed. Usually, WfMS' provide a single centralized workflow scheduler with a centralized networking model (e.g., web-server to clients), which is not the ideal solution for executing a scientific workflow as its network layer has to deal with a high rate of control messages. Thus, even if only control messages are exchanged, it can become a bottleneck and misuse the network capabilities offered by the grid. In our scenario, nodes exchange data by following a choreography perspective, whereas control flow is set up using a special component we call Coordinator. The use of this kind of solutions is already present in previous works [10], where Coordinators monitor the performance of the SWf and bind the inputs/outputs of tasks with the underlying Pub/Sub network and identifiers.

### 2.2. Workflow Model of SWf

We define a workflow model based on tasks and activities that are allocated in local or remote nodes and mapped to the underlying Pub/Sub event layer. This SWf model evolves from our previous work [20], its service foundations and concepts of tasks and activities. A workflow consists of the set of logical tasks and the communication channels between them, which are supported by a processing infrastructure on top of the event layer. Workflows are executed in a distributed way and logical relationships among tasks, which represent their internal behavior, are arranged in the fragmentation or partitioning process [22]. The fragmentation process covers the actions of computing, initializing and distributing a set of tasks. Tasks are logical fragments of the workflow executed in local or remote nodes. In the SWf execution aspects such as elastic scalability, lifecycle management, security must be considered, but they are beyond the scope of this paper. In order to focus on our models, we assume that mechanisms exist to place and create tasks instances, so tasks are executed by a module called Orchestrator [20], which is present in each node that participates in the SWf execution and an underlying middleware [15] that provides Pub/Sub protocol support. A task is composed of at least one Activity. An *Activity* is an atomic unit of a task that has inputs and outputs. It manages the communication with an object that can be physical or digital, in order to perform an operation. For example an activity can be the action of requesting a local database value, or a remote notification of a finished job. Activities can trigger control events *(ev)* and consume those ones produced by activities running over different tasks. Therefore, from the communication perspective they trigger the *publish(ev)*, *subscribe(ev), and un-subscribe(ev)* primitives of the Pub/Sub network and are *notified(ev)* by brokers. We define *Limit Activity (A_L_)* as any activity that communicates with other activity contained in a remote task, so *A_L_* can act, in runtime, as a producer, consumer or both. A control event is the action of transmitting a control message (e.g., to start the *A_L_* execution); however, we use them as similar term when referring to our communication model.

**Figure 1.**
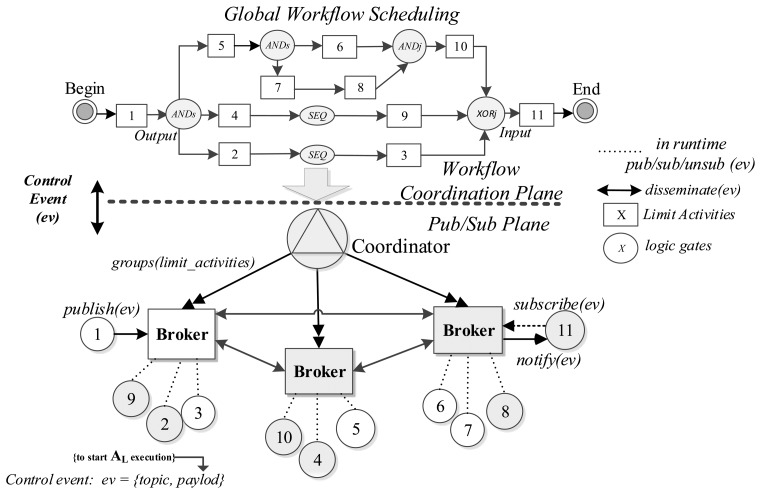
Workflow-to-Pub/Sub mapping.

We use logic gates to enable communication between tasks, in the control plane. These logic gates follow the patterns model, defined by Van Der Aalst *et al.* [19], corresponding to basic control flow patterns, advanced branching and merging. Thus, hereinafter we use workflow pattern and logic gate as the same concept. Figure 1 illustrates the two different planes that our model targets, from the global scheduling perspective of the SWf. It also shows how control messages are mapped to Pub/Sub primitives and events and later disseminated over a distributed broker scenario. Limit activities are linked by logic gates, which in turn trigger control messages that activate subsequent ones.

## 3. Supporting the Scientific Workflow

The following sections explain the models we propose to support a decentralized SWf execution as well as the workflow messaging. Hence, our objective is to improve the workflow coordination, at the event level, by leveraging the complexity of the dissemination of control events to brokers, and transfer to them the interactions among remote *A_L_s*. At the end of this section, we provide an example regarding the support of our SWf model including the initialization and runtime over the Pub/Sub layer.

### 3.1. Supporting the SWf

The proposed model is composed of the definition of a task interoperability reference model, the mapping of workflow activities to Pub/Sub topics, the interfaces that allow setting up the Pub/Sub layer, the broker reference architecture, and finally the procedures for dealing with binding recovery between activities in runtime.

#### 3.1.1. Task Interoperability

Conceptually, tasks must agree on the control messages their *A_L_s* generate or require, and how they are mapped to control events in the Pub/Sub layer. Therefore, brokers have to filter and only deliver those ones that match subscription requests. To do this we use a topic-based Pub/Sub language [23]. Messages are published using “topics” that identify *A_L_*s' outputs and subscriber *A_L_*s subscribe to topics representing their triggering condition or inputs.

In order to define a common hierarchy of topics, we use a topic domain shared by all tasks and divided into namespaces. Topics are published in namespaces in order to receive control messages in the appropriate language and ensure that only compatible events are pushed by brokers. Each topic in a topic namespace (*tns*) can have zero or more child topics and a child topic can itself contain further child topics. A topic without a parent is termed a root topic. We use the forward slash (/) character to indicate a “child of” relationship. For example, the *tns1:monitor/exception* refers to the subtopic exception, subset of the parent topic monitor, in the namespace *tns1*.

This approach supports transformation and aggregation of topics. It is possible to construct configurations (using intermediary brokers) where the topic, an interested “subscribes to” differs from the topic under an entity “publishes”. Thus, the broker, acting in line with administratively-defined rules, receives the control messages from the publisher, matches and notifies the corresponding subscriber. For example, a subscriber to the topic *tns1:monitor* also receives notifications from topic *tns1:monitor/exception*. It is possible for participants of the SWf to define additional topics based on existing topics without requiring coordination with the participant responsible of creating the topics that are being built on. Our solution is compatible with the WS-Topics OASIS standard [24], which presents a set of “items of interest for subscription” in Web service environments, and it has been extended to be aligned to a non-WS environment.

**Figure 2.**
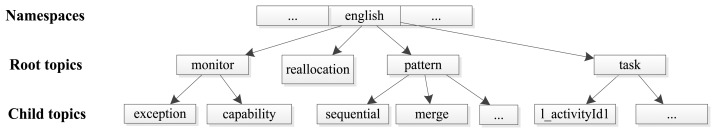
Topic Hierarchy.

An example of a topic hierarchy for a generic SWf is shown in Figure 2. This *tns* corresponds to the English language, to avoid language incompatibilities. To prevent correlation problems, a root node has been added, so it contains the identifiers of the task instances that are being executed.

#### 3.1.2. SWf Initialization

**Figure 3.**
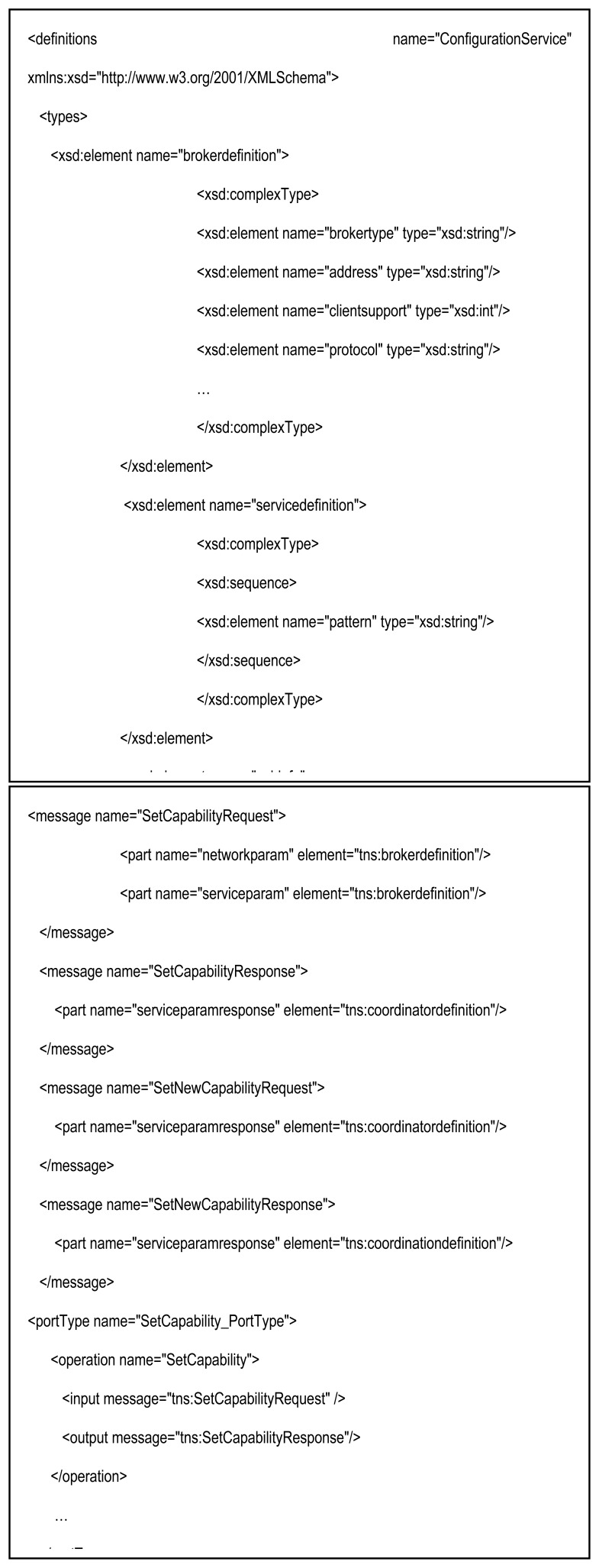
WSDL interface.

The workflow initialization over the Pub/Sub events requires an interface between the Coordinator and brokers. This interface implies the loading of Web Service Description Language (WSDL), which is defined in this section. Thus, prior to the execution of the SWf over participants, it is necessary to link the logical interest of an *A_L_* (*input or output*) to the communication primitive that will support it: *subscribe(ev)* or *publish(ev)*. In other words, it is necessary to bind the SWf plane with the Pub/Sub layer and comply with the logical gates that join tasks and their *A_L_*. The initialization process refers to mechanisms that support the binding of an action of a predecessor *A_L_* with a Pub/Sub event, the associated logic gate, and a subsequent reaction of a successor *A_L_*. Therefore, as the Orchestrator of each participant detects the *A_L_* s that makes up part of the task instance, the topics for each input and output *A_L_* must be designated. For this task, we use the Coordinator function. From here onwards, the Coordinator registers the references of predecessor and successor of every *A_L_* of the SWf and generates the topics identifiers by following the namespace previously proposed. In this process we assume that the Coordinator have already received the initial SWf structure and logical relationships between activities from a global SWf scheduler or a workflow composer (e.g., the database created by the Trident SWf composer [25]). Brokers inform the Coordinator about the network capabilities they support, such as supported protocols, and the set of logic gates they can instantiate. Hence, the coordinator can know, in advance, which of the brokers can instantiate a logic gate and link corresponding *A_L_*s. Then, as each *A_L_* has its own topic the Coordinator can group these activities using the initial SWf structure. The Coordinator sends to brokers the information regarding *A_L_s* that produce events (predecessors) and the *A_L_* that are triggered by these events (successors) over the same SWf instance. This information also includes the callback addresses of Orchestrators that are executing the corresponding tasks. Afterwards, brokers use this information to internally group subscriptions using the model explained in Section 3.3. In order to maintain a generic and flexible coordination interface between brokers and the Coordinator we define the WSDL shown in Figure 3, which follows the same concepts of current SWf systems such as Trident [25].

The WSDL describes the logic gates (or patterns) types, and relationships between *A_L_s* they enclose. The *SetCapability* field is used by the Broker to express its capabilities to the Coordinator, whereas the Coordinator makes use of the corresponding response to set the predecessors and successor *A_L_* in the workflow initialization. *SetNewCapability* messages are sent, by the Coordinator, to brokers with the objective of grouping logic gates and *A_L_*s.

The Coordinator groups activities depending on the predecessor and successor relationships between *A_L_*, so it puts them, by default, in the same broker. In the case this process is unfeasible (e.g., due a network constrain or a request of the SWf manager), the Coordinator can set a successor activity in a different broker than its predecessor. This one of the cases we show in Figure 1, where *A_L_-4* and *A_L_-9* are predecessor and successor, respectively, and are set in different brokers. In this situation, the Coordinator marks the predecessor callback string as “*remote*”. Then, it attaches in the *predecessor* field of the message specified by the WSDL, the network address of the broker that supports its predecessor *A_L_* followed by the topic that identifies it (e.g., *remote,http://*<*x.x.x.x*>,/*task/activity1*). Therefore, the broker of the successor *A_L_* subscribes to the broker of the predecessor *A_L_*., and gets ready for receiving control messages that target the successor *A_L_*. This type of recursive subscription has been well-proven over the Internet and many Pub/Sub protocols supports them (e.g., PubSubHubbub [26]), so our solution can be considered independent from any specific implementation and remains compatible with our previous research [21] focused on simple and scalable gossip-based interactions. Our model is also independent from the specific protocol used to communicate brokers and the Coordinator, as long as the WSDL interface is used.

#### 3.1.3. Binding Control Events in Brokers

**Figure 4.**
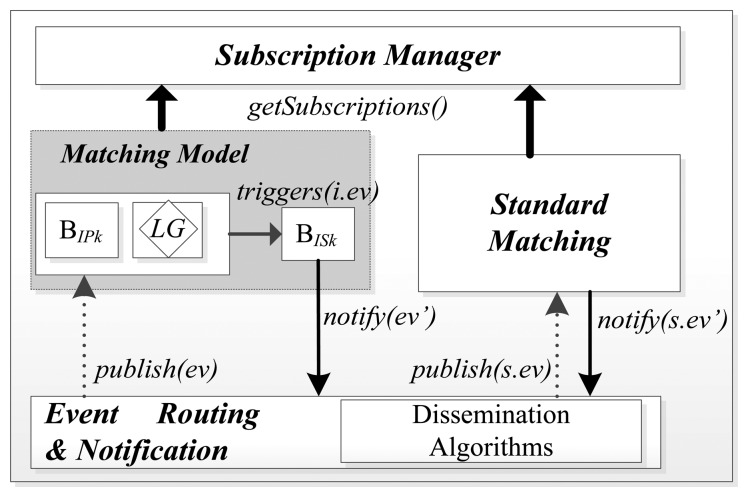
Reference Broker Architecture.

As previously mentioned, our objective is to delegate to brokers the complexity of communication between *A_L_*s and logic gates. The broker reference architecture used to support workflow patterns is similar to standard topic-based brokers. The broker registers subscriptions from clients, matches control events and disseminates these events to subscribers or other brokers. Workflows are supported using a pluggable matching model which works on top of standard broker processes. Hence, while standard topic-based brokers match incoming events and notify the right subscribers based on their interest, our broker firstly filters the control events that fulfill the logic gates and later notifies subscribers. So, even if successor *A_L_*s subscribe to a task and their predecessor subscriptions publish a control event, the broker dynamically holds the notification of this event until the logic gate is satisfied. The notification is dynamic because the broker can react to changes in the relationships between activities which were extracted from the WSDL as we explain in Section 3.1.4. Our broker enforces the patterns following the models described below. Having this architecture, our broker is capable of decoupling *A_L_*s, their role and actions they can trigger in other workflow branches or tasks executed in parallel (e.g., an activity that calibrates time). The matching model performs as a pluggable component as it only needs to internally receive all the published events from every *A_L_* of the WSDL, in order to correlate and notify the correct event. It can work on top of standard topic-matching functions without any disturbance or special synchronization. Figure 4 shows the reference broker architecture.

We use the term internal binding (B_I_) to describe how we model the relationships between *A_L_* and logic gates inside the broker. LG represents the process of enforcing these gates in runtime. We consider our model a composite binding of two internal bindings. We apply the term external subscriptions (S_E_) to describe the relationship between a topic, which represents the interest on the LG fulfilling a control event, and a callback address of the Orchestrator that runs the *A_L_*. In our model, as the S_E_ implicitly fulfill the two bindings a standard event notified to a subscriber is defined as: *s.ev'*=*f(s.ev,S_E_)*. When we add the logic gate component, this event is defined as follows:

(1) $$ev'=f(\sum_{i}^{k}(B_{\text{IPk}}),\sum_{i}^{k}B_{\text{ISk}},LG)$$

where B_IP_ and B_IS_ define bindings with predecessor and successor (S_E_) respectively. Being B_IPT_ and B_IST_ the whole set of predecessor and successor subscriptions: *B_IPK_* ⊆ *B_IPT_* and *B_ISK_* ⊆ *B_IST_*, where B_IPT_ and B_IST_ are the binding spaces for a given task instance. Then, the difference between matching an event using standard matching and our matching model is enforced at this point. Whenever a *A_L_* generates a control message that is published to the brokers, in the case of standard matching, the evaluation and notification of the event is straightforward since the broker only ensures that the event's topic corresponds with the existing S_E_. Nevertheless, in our model, whenever a similar control message arrives to the broker, it broker performs (depending on the pattern) the latter performs the steps described in the following paragraph. Figure 4 depicts the broker reference architecture.

In runtime, a predecessor binding is an instance, inside the broker, subscribed to a control event an *A_L_* triggers. In the case that this event fulfills this binding; the broker evaluates the Logic Gate (LG) where B_IP_ belongs and triggers an internal event (*i.ev*). Then, the broker matches this event with the successor bindings which contain the real subscribers' callbacks of the logic gate, and *notifies(ev)* them. Retaking Figure 1, the input of *A_L_*-9 is represented by a B_IST_, and the output of *A_L_*-4 by B_IP_; so in order to modify/activate the execution of *A_L_*-9, the broker evaluates if there is a B_IP_ interested in the output event produced by *A_L_*-4. Next, as it is true and a Sequence flag is active, it generates an internal event captured by B_IST_, which represents the interest of *A_L_*-9; then, the broker pushes the control message to the callback address of the Orchestrator. As we already mentioned, in our model we support six workflow patterns, which are the basic control-flow patterns that are used to build workflows. The implementation mechanism proposed in this paper focuses on the six selected workflow patterns, as they are the most elementary of the entire existing workflow pattern and are used for building more complex SWF. Following this approach, now we explain how we support each case assuming that each of the activities we mention are limit ones.

*Sequence (SEQ)*: it is the pattern we used to explain our model right before. In this pattern, a single activity is enabled after the completion of the preceding activity. Thus, brokers establish a one-to-one relationship between B_IP_ and B_IS_; so, B_IP_ always triggers an *i.ev* that leads to the real subscriber and its activity.

*Parallel split (ANDs)*: in this pattern, a set of activities are enabled after the completion of the preceding activities. Brokers establish a one-to-many relationship with B_IP_ and many B_IS_. Bindings that represent the successor activities are triggered by the same *i.ev*, however, unlike the sequence pattern; the broker matches the *i.ev* with the successor bindings they are interested in. This operating mode is due to B_IP_ and B_IS_ can be part of other patterns, so keeping an internal reference among them, allows decoupling the particular activation of the LG instance from other relationships or interests internal bindings can have.

*Exclusive choice (XORs)*: having a set of candidate activities to be enabled; only one is enabled after the completion of a prior activity. In this pattern, brokers also establish a one-to-many relationship with B_IP_ and B_IS_s. The pattern depends on the control event that defines which activity must be enabled, so, we model this event as: *c.ev* = *f(B_LG_, ev)*. As we are using a topic-based language, this event contains the topic of the B_IS_ that follows the workflow.

*Simple merge (XORj)*: this pattern defines the convergence of two or more activities into a subsequent activity. The broker establishes a many-to-one relationship with B_IP_s and B_IS_ respectively. Since no synchronization is needed, whenever the first B_IP_ is matched with a control event, the LG lets this event reach the B_IS_ as an *i.ev*. This process is available for any of the B_IP_. After each process, the LG is re-initialized in order to support new matched events arriving from the same B_IP_ or new ones.

*Synchronization (ANDj)*: this pattern defines the convergence of two or more synchronized activities into a new activity. In other words, in order to enable the subsequent activity, all the previous activities must be enabled. This pattern is modeled as a many-to-one relationship with B_IP_s and B_IS_. In this pattern we do not take into consideration correlation issues [27], because we assume that brokers can implement buffers, timestamps or any other mechanism to address them. We start from the fact that brokers receive the correct events. Hence, every time the broker enforces the sync pattern, it links each predecessor activity to an open lock, so every time an event satisfies a B_IP_, the broker closes its lock. Next, in the case that all the activities are locked, the *i.ev* is triggered and the broker verifies the covered B_IS_, so then, it informs the corresponding task's *A_L_*.

*Multi-choice (ORj):* this pattern describes the divergence of an enabled activity into one or more activities, so the execution of the successive activities is enforced using a dynamic condition. The way LG supports a dynamic condition is through the same control event of the exclusive choice pattern. Thus, once the LG is enforced the broker triggers an *i.ev* that reaches the B_IS_ and activates the corresponding activity. At this point, the challenge consists of how to allow different B_IS_ to only consume *i.ev* under the conditions determined by the received control events. Assuming that these conditions include the identifiers of each subsequent activity, our solution consists of letting B_IS_ to internally subscribe to their same instances. Then, *i.ev* messages targeting these identifiers will be created and the matching process will be ready to verify if the event satisfies only the enabled B_IS_. This strategy is feasible using topic-based languages (because the same topics can be used as identifiers) and non-distributed subscriptions (as the cost of producing events is lower).

#### 3.1.4. Workflow Recovery Support

**Figure 5.**
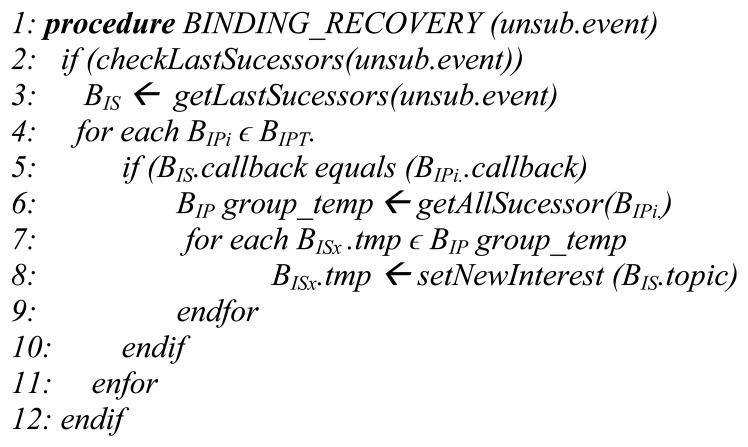
Binding recovery pseudocode.

In our model brokers are capable of updating internal subscriptions in order to avoid unreachable states or inconsistences in the SWf execution. The sequential pattern is the most elementary supported logic gate. Remaining logic gates can be modeled as a sequential pattern in the case of having only one predecessor and one successor activity. A distributed workflow (using many different logic gates) can experience this kind of situation because of on-demand actions or due to runtime inconsistences. In order to support these actions and prevent inconsistences and deadlocks, we propose the pseudocode shown in Figure 5. It recovers and reconnects predecessors and successors bindings that trigger the real events pushed to limit activities. This method is feasible because brokers previously received all the relationships between activities with the WSDL. It is compatible with alternative limit activities that could appear in runtime because each time an un-subscription (unsub.event) occurs the broker recovers the same relationships that are employed in the matching.

As an example a SWf is composed of instances of tasks A, B and C which are linked by sequential activities in the same order. Hence, task C consumes events produced by task B and the latter from task A. The pseudocode uses as input, every un-subscription event received by the broker. This un-subscription represents a changing state in tasks and therefore in its *A_L_*s. The un-subscription can be triggered by on-demand actions of the SWf scheduler, or by an event of unexpected disconnections in implementations that are based on ping-based interactions (e.g., WebSockets ping). Step 2 checks if there are still successor bindings (in this case inputs targeting tasks B) interested on the events produced by task A's activities. In the case the un-subscription event corresponds with the last remaining successor binding that is interested on events received from task A, the algorithm recovers (Step 3) the successor binding. Next, the algorithm iterates (Step 4) each of the existing predecessors bindings and then compares if the callback address of B_IS_ corresponds with any callback address that has published under the identifier of *B_Pi_*. In this process the topic can be used as this identifier, as we maintain a strict hierarchy, but it is still compatible with other mechanisms. Afterwards, Step 6 searches for every successor binding, which represents in our case the input for the activity of task C, willing to consume the activation events *(i.ev)* resulting from the gate's enforcing. This step can be compared with a membership test which is well-supported using fast matching techniques (e.g., through Bloom Filters [28]). At this point the remaining step consists on recovering (Step 7) each one of the successor bindings and set them with a new interest that will be at the end, the triggering input of the successor activity of tasks B.

The algorithm targets subscriptions that are being supported in the same broker, nevertheless in the case the broker ends its execution and no new interests are set, being the distributed nature of the SWf, it can execute a mechanism [29,30] to disseminate these disconnection events. In the case of synchronizing patterns it contributes to the self-healing characteristics of a SWf as tasks depend on the correct scheduling of every one of the previous branches tasks and their *A_L_s*. As a final point, the pseudocode takes as triggering points the un-subscription actions that enclose successor binding instead of predecessor bindings and this characteristic is because it targets SWf based on directed acyclic graphs.

### 3.2. Workflow Messaging

**Figure 6.**
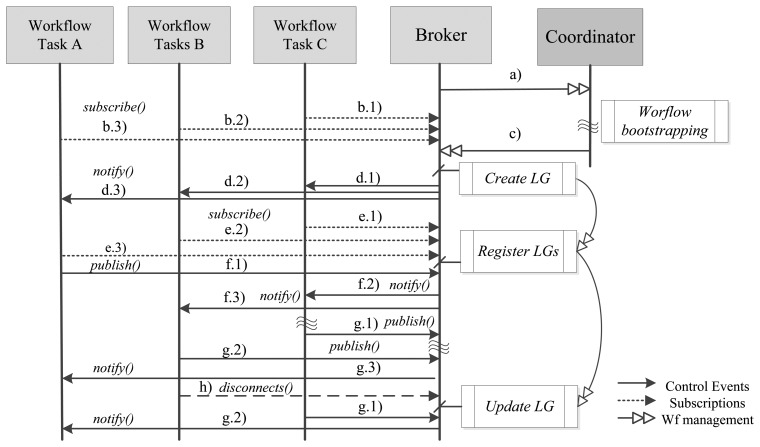
Workflow messaging.

In this section we explain how our SWf model behaves in the Pub/Sub layer in initialization and runtime, as it requires some primitives in the event layer *(publish,subscribe,notify,un-subscribe)* in order to bind tasks and their limit activities. Figure 6 shows an overview of the workflow messaging message exchange among tasks A,B and C, one serving brokers and their Coordinator. In the global schedule model, these tasks carry on the *A_L_s* we showed in Figure 1. Tasks A, execute *A_L_-5* and *A_L_-10*. Task B executes *A_L_-7* and *A_L_-8*. Task C executes *A_L_-6*.

In the first step the broker that will support the SWf participants sends (a) to the Coordinator its capabilities using the WSDL description presented in Section 3.1.2 Workflows' participants subscribe (b.1,2,3) to the broker and wait until topics corresponding inputs and outputs of *A_L_* are assigned. Once the Coordinator initializes the topic identifiers of each *A_L_*, it pushes this information to the broker (c), which matches the correct workflows participants using the client identifier it has received from the Coordinator. At this point the broker creates the internal bindings, by arranging predecessor and successor activities, and uses the callback information to notify (d.1,2,3) tasks A, B, and C with the topics identifiers that represent these *A_L_*s.

The first step towards the global execution of the SWf is to inform each one of participants regarding the topic identifiers. Once tasks subscribe (e.1,2,3) to the broker, it registers these subscribers as successors and gets ready to notify control messages. We developed logic gate cases of the *ANDs* and the *ANDj* as they encompass, in terms of message exchange, the remaining gates. These two cases involve many-to-many communication and control messages that trigger activation of branches activities. In the *ANDs* case, *A_L_*-7 and *A_L_*-6 are the successors; so whenever tasks A reaches *A_L_-5*, it publishes an event (f.1), using *tns:pattern/parallel/cevent*, and the broker notifies (f.2,3) them. Following the SWF execution, activities *A_L_*-8 and *A_L_*-6 are the workflow predecessors and *A_L_*-10 the successor. They are connected through an *ANDj* pattern, so it requires that all the predecessor activities must be completed before the execution of the successor one. Following the binding model of Section 3.1.3, the broker implements the LG and marks *A_L_-6* and *A_L_-8* as B_IP_s, and *A_L_-10* as B_IS_, so the broker will only trigger the *i.ev* if both B_IP_ are satisfied. After the execution of *A_L_-6*, it publishes an event (g.1) that matches the first B_IP_. At this point, the LG conditions are partially fulfilled as the broker retains the event that triggers the B_IS_ and the activation of *A_L_-10* until Participant *A_L_-8* publishes (g.2) the corresponding remaining event that will match the remaining B_IP_. After any given time, if Task B disconnects from the broker (e.g., due to a planned action or an unexpected situation such as a network failure), the latter updates the predecessor binding registry for this LG. Hence, in the case *A_L_-6* generates a new event; the broker directly matches the only existing successor binding and triggers the control event targeting *A_L_-10*, as there is no need to wait any other predecessor binding. Participants can also un-subscribe to topics, by issuing the primitive unsubscribe (topic) and the broker will enforce the process explained in Section 3.1.4.

**Figure 7.**
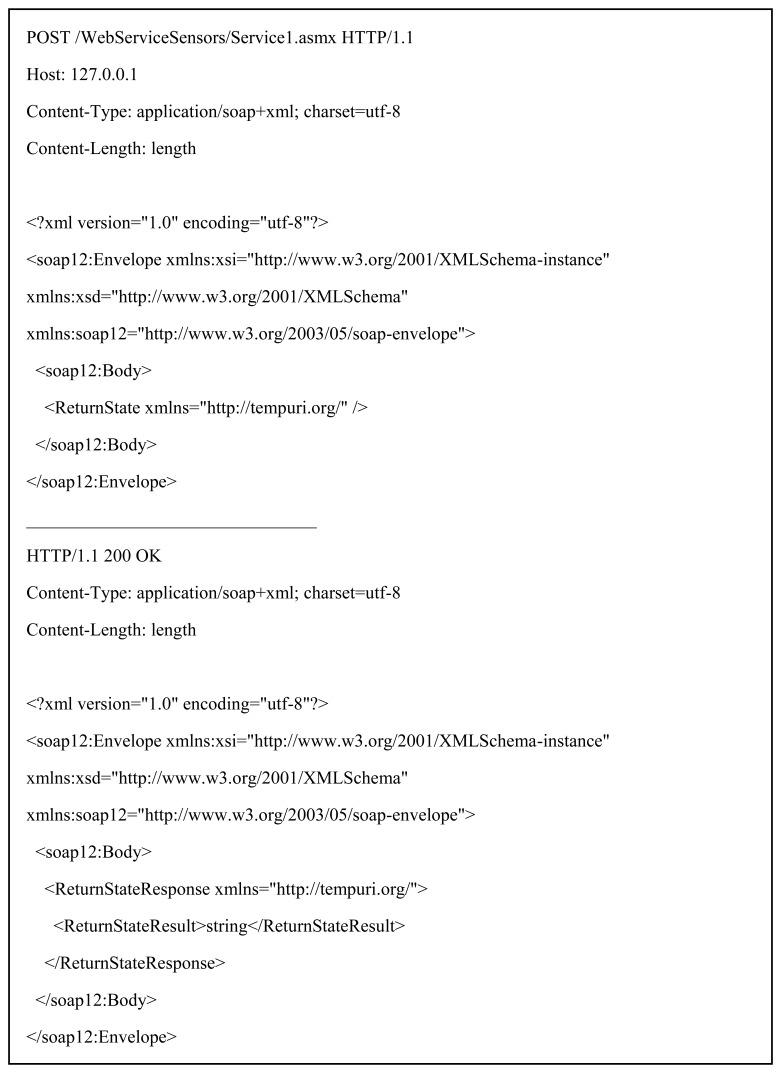
Example of SOAP request and response.

In the case of a broker failure the Orchestrators use a pre-configured backup broker and publish the network properties of the failing broker using the topic: *monitor/exception/broker*. Therefore, as the Coordinator is already subscribed to this topic, it will receive this information. Afterwards, it confirms the broker status by issuing the SOAP request shown in Figure 7. Both negative and positive responses will be informed to the overall SWf Scheduler. In the negative case, it will be caused by a connectivity issue between the participant and its broker. The second one represents a total failure of the broker, so the SWf will need a reallocation of participants to an available broker. Specific network recovery model and re-scheduling mechanisms are out of the scope of this paper, so we assume that Coordinator can always reinitialize the SWf using the process explained in Section 3.1.2.

## 4. Communication Model and Application Scope

As we stated, our model is based on the principle that workflow patterns are supported in brokers, rather than in nodes. Therefore, we have reviewed in the literature existing systems that support the execution of SWf, and then we have classified them into three communication solutions based on how they support the control message exchange. Then, the objective of this section is to analyze the expected requirements of SWf execution in the context of workflow patterns and in-runtime message exchange. Later, we use this analysis to extract the advantages of our proposal in comparison with the other three communication solutions. These requirements are: information dissemination, info dispatching and filtering, task decoupling through workflow patterns, flexibility over workflow changes, scalability of events and workflow monitoring and failure handling.

**Figure 8.**
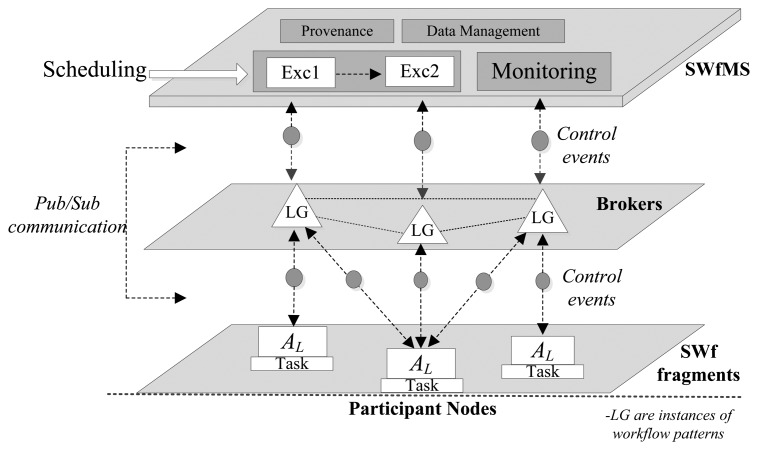
Relationship between patterns, participants and events.

In this section we demonstrate that offering workflow pattern support, at the message exchange level, offers more global advantages to SWf participants in terms of information delivery, task decoupling and so on. Afterwards, we provide a quantitative evaluation of the solutions. Figure 8 shows the relationships between the workflow patterns, participants and control events.

The first solution is based on a semi-centralized messaging (SCM) in the SWf scheduling; in this solution, workflow patterns that connect tasks are supported over centralized schedulers or a pool [31] of decentralized ones under the same SWfMS. Therefore, we can assume that communication between different tasks is supported by establishing channels between tasks and schedulers, no matter the underlying characteristics of the dissemination layer (e.g., a tailored grid [6,7,11,32] or web-based messaging [33]); this characteristic is because the workflow scheduling of tasks is still linked to control events triggered by schedulers in response to the workflow patterns. Hence, from the underlying communication perspective, these control events are stored and forwarded by only one entity (e.g., a built-in or a remote message broker).

The second solution is a distributed scheduling messaging (DM) between tasks. Tasks are scheduled [34] to run in a distributed table over a P2P-based scenario, which is shared among participants, rather than in a cluster or a pool of schedulers. Triggered events are decoupled from schedulers and the running conditions (which also depend on workflow patterns) reside in the same table in the form of states (pending, running and finished).

We call the third solution Pub/Sub with channel optimization messaging (COM). It is based on our previous research [20] and inherits its mechanisms for establishing channels associated to task and the support of workflow pattern in the same nodes that execute these tasks. Therefore, nodes running tasks are loosely coupled from schedulers in execution, at expenses of dependency in space. Finally, we name our whole solution model as Bounded patterns over Pub/Sub (BPoPS). A summary of the comparison is depicted in Table 1.

### 4.1. Qualitative Comparison

*Information dissemination* refers to the mechanisms that allow tasks to gain access to the branch conditions via control messages. In the SCM solution Orchestrators executing tasks are always coupled to other Orchestrators in terms of location of the target tasks (space coupling) and the flow interaction dependencies among them (synchronization decoupling). This fact is a disadvantage for the SWf as its execution is dependent to this coupling no matter the message exchange pattern: many-to-one (e.g., *ANDj*) and one-to-many (e.g., *ANDs*). The DM solution provides a well-proven information dissemination environment as tasks can be fully-decoupled from the scheduling systems. However, maintaining a shared, synchronized and consistent information space between a wide range of SWf participants, and their intermediary patterns [35], leads to networking overhead and complex scheduling datasets in comparison with the COM and BPoPS solutions.

In BPoPS we leverage to brokers the scheduling order and the triggering conditions generated by activities bindings in runtime; so tasks remain agnostic of the other tasks with which they have no runtime relationship. Regarding sync decoupling, as brokers carry on the message storage and forward functions, participants are set to be straightforward and lightweight (from the dissemination stack perspective). Hence, this model offers better capabilities as tasks get control messages *over a fully asynchronous and decoupled communication model*, while the global SWf scheduling *remains compatible with event dissemination mechanisms* (e.g., Gossip-based [21]) targeting distributed networks and grids.

*Message dispatching and filtering* refers to the mechanisms for creating, dispatching and later filtering of control messages. In the SCM messages directly reach the right participants' Orchestrators. Whenever a scheduler jumps from one task to another, the Scheduler decides which of the task participants it needs to trigger based on the workflow patterns. The process of pushing messages is carried out at the Application Level, as the global scheduler is aware of the network location and information models of the successor activities. This mechanisms offer advantages for centralized SWfs, because no overlay or intermediary functions are needed, however, it is proven that a centralized information delivery is not the best method [17] for delivering messages in SWfs.

In the DM solution, the overlay network filters workflow control conditions. Depending on the network topology (e.g., pure and hybrid P2P), the schedulers can directly coordinate the execution (point-to-point). Tasks are registered to predecessors and successors tasks using recursive P2P-based searches that act as message filters between them. These kinds of methods require [36] complex scheduling mechanisms in order to balance the load and delivery messages in contrast with our BPoPS solution were *messages can be filtered at the nearest point* such as the tasks' broker.

In the COM solution messages are filtered and forwarded to participants that execute tasks, however, it produces a high level of long-lived subscriptions between nodes and schedulers, which makes this solution unfeasible for complex and distributed SWfs. In the BPoPS solution, brokers maintain the forwarding and event filtering level because they filter the events using topics that represent bindings under administratively-defined rules. The BPoPS solution *enhances the event interoperability with SWf task instances that could be added in runtime* (e.g., a new set of optical sensors) as brokers enforce an Event-level filtering based only on topics which are independent from the information models used by activities.

*Task decoupling through workflow patterns* is a characteristic that allows SWf participants to maintain decoupled the relationships between tasks. Synchronized communication provides limited task decoupling, which is non-expected characteristics for distributed SWfs [3]. In the asynchronous SCM case the SWf execution is dependent on the blocking conditions and synchronization generated by scheduler as it is the only capable of retrieving successor activities in the whole SWfMS. This limitation applies no matter what state the task is in or the pattern that joints its activities.

In the COM solution tasks acting as publishers and subscribers are partially decoupled. This is because the *XORj* and *ANDj* patterns obligate successor activities to subscribe to each one of the predecessor activities. In addition, as each one of the subscription to these activities establishes a single and tailored channel (that can be compared with a network address), this solution breaks the principle of Pub/Sub communication regarding space decoupling.

The BPoPS solution is the most complete one, as it fully supports tasks decoupling since A_L_s can subscribe to other ones using the same topics, regardless of their binding pattern. Our proposal is also compatible with the process of adding and removing A_L_s (using the WSDL) by schedulers and allows other related tasks to continue their execution without disturbance in the case of SWf changes; for example by executing the pseudocode presented in Figure 6. It is not illustrated how the DM based model can afford the task decoupling thought workflows patterns.

*Flexibility* is the ability to adapt to SWf changes. In the SCM case the flexibility is limited by the amount of activities-to-activities bindings; so schedulers must be capable of re-ordering the SWf execution in the case of changes in the execution order. In the SCM solution, schedulers must contact each one of the SWf tasks, so it limits the workflow to a centralized point of failure and decreases the SWf flexibility. The DM offers high flexibility over network changes as it depends on single links between schedulers and tasks participants. The COM solution embodies similar flaws than the SCM solution in the case of *XORj* and *ANDj* patterns since the successor activity must be aware of the state of its predecessor *A_L_s*. Therefore, this scenario obligates tasks to be subscribed to all of these outputs, which increases the messaging and the implementation complexity of the Orchestrator of the predecessor activity. In our solution as the entire branch conditions pass through brokers, *they can implement mechanisms to recover the workflow bindings and dissemination protocols (*e.g., *gossip-based) that complement the network flexibility and participants' simplicity*. Depending on nodes, the workflow scheduling and the scenario where it runs (e.g., structured SWf topology and lightweight participants), our solution offer suits more qualitative advantages as we state in Section 4.2.

In our context, *scalability of events* is the increase in the number of total connections among *A_L_s*, so the less connections the SWf execution requires the more scalable it will be. In the case of the SCM solution connections grow in proportion to the number of *A_L_s* added to the SWf, which makes this solution costly in terms of networking resources. In the DM solution tasks fetch the execution stages from shared information, so they can directly communicate with other tasks. Therefore, scalability depends on the number of participants being part of the SWf rather than the running conditions. As SWfs can be very dynamic in runtime the DM solution offer less advantages that our proposal in static networks. The COM case uses Pub/Sub communication, which has been proven its good scalability [3,37] delivering control messages in workflows. Nevertheless, in the case of having a SWf with a large number of events and joint-based patterns (e.g., *XORj)*, as these brokers are agnostics in terms of patterns, the task (and its successor *A_L_*) must be subscribed to every one of predecessor *A_L_*. So, unnecessary messages will reach tasks and decrease the scalability level of the whole SWfMS. In our solution *connections grow with changes in the workflow running conditions, which required requires less messaging because no unnecessary control messages will be pushed to tasks*.

*Workflow monitoring* refers to the process of verifying that the workflow execution conveys with the messaging that leads to the correct ordering of *A_L_* execution and no errors, deadlocks or inconsistences occur. In the SCM solution if the number of participants increases, having a centralized communication (e.g., from tasks to schedulers) offers a lower decentralization level in comparison with other communication solutions [3], and bottlenecks may occur as well. This disadvantage is because schedulers have to keep track of every state the whole workflow has gone through, in order to handle a failure by for example informing the right successor *A_L_*, or re-scheduling tasks' *A_L_*s. We consider that the DM solution offers higher monitoring capabilities as the state of task is distributed in a shared space and inconsistences are accessible by schedulers.

The COM solution introduces the same limitation inherited by the scalability of events. The BPoPS solution allows the monitoring system *to save network and processing resources*. Brokers act as the monitoring entities of tasks, because they already serve them and control their bindings with other tasks' *A_L_*. They can properly deal with failures (e.g., by applying the algorithm of Section 3.1.4.) and inform schedulers about the state of tasks using the topic hierarchy presented in Section 3.1.1.

### 4.2. Evaluation of Communication Solutions

In this section, we describe a validation scenario for the qualitative evaluation of communication solutions described in Section 4.1. This validation scenario presents a realistic situation of the implementations of the proposed solutions according to the use that is made today of the Internet applications and services. It has been designed for supporting the runtime behavior of the SWf described in Section 2.1. Building this scenario we provide an integral evaluation of the communication solutions including a quantitative comparison based on the performance of each communication solution this scenario. Henceforth, we use the same SWf showed in Figure 1 as the input for all the implementations.

**Figure 9.**
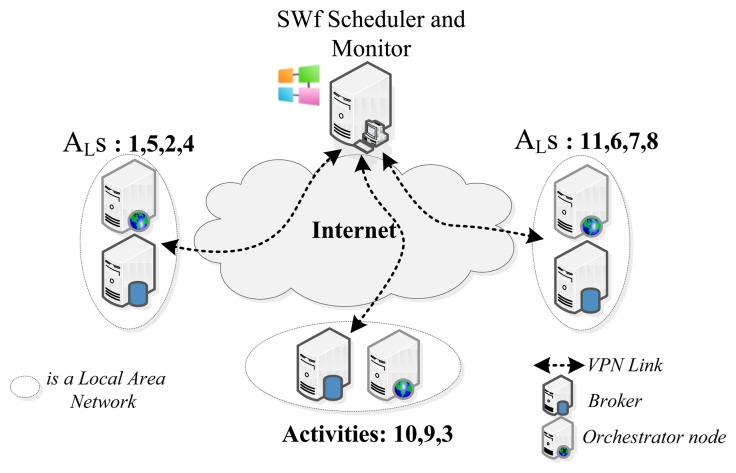
Network topology of the evaluation scenario.

The validation scenario of this SWf consists on a set of A_L_ deployed over the Internet in a distributed manner and well-defined and structured administrative domains where: IP multicast is not available, predecessor and successor A_L_s are supported by a Orchestrator node and communicate with the SWf Scheduler/Monitor (in our case the Trident Workflow Workbench) using a Virtual Private Network (VPN) channel, A_L_s bindings and their message exchange are maintained in a private domain and finally, A_L_s are set to be in a static local area network with low addressing movement. Brokers communicate with Orchestrators and the SWf Scheduler and Monitor. Figure 9 shows the network topology of the evaluation scenario.

As we analyzed in Section 4.1, DM-based solutions are suitable for scenarios where SWfs have a high level of re-scheduling, parallelism and dynamic peer topologies. In the evaluation scenario, there are static relationships between peers, in terms of network addressing, and a low level of re-scheduling; therefore: (i) a DM-based solution offers qualitative characteristics that are barely applicable to the evaluation scenario but increase the complexity of SWf management and its implementation. In addition, (ii) the evaluation scenario comprehends administratively-defined and controlled domains that are difficult to maintain and support using a purely distributed P2P approach in current SWf frameworks. Concerning the relationships between predecessor and successor A_L_, DM-based solutions concentrate on delivering data to peers (through P2P paths) and resolving connectivity issues in an unstructured network. The evaluation scenario is set to be in a private and structured network domain, in terms of exposed interfaces, addressing and notification of control events that realize the bindings among predecessor and successor A_L_. Therefore, (iii) even with secure path selection and routing mechanisms, a DM-based solution is less suitable for the evaluation scenario and overheads the communication stack of nodes supporting Orchestrators. In the context of the evaluation scenario and taking into account these three implementation drawbacks of a DM-based solution, we clearly state that its implementation offers disadvantageous qualitative aspects in comparison with the other solutions analyzed in this article: SCM, COM and BPoPS. Therefore, we have focused our efforts into providing a quantitative comparison among SCM, COM and BPoPS implementations, which share the same qualitative characteristics expected for this evaluation scenario.

In order to normalize the comparison and overcome the differences that can be originated by different implementations and communication models, we set up a common layer for the three implementations. This layer is based on SOAP-HTTP. As we analyze the runtime behavior, we assume that the SWf was previously initialized including the information regarding A_L_' bindings, predecessor/successor A_L_, broker addresses and logic gates.

We used the Trident Workflow Workbench [25] as the main framework to *compose, schedule* and *monitor* the SWf execution. It ran over a workstation with a Core i7 1.6 GHz CPU, with 4 GB of RAM, and the 64 bits Windows 7 OS. We started from the fact that A_L_s were already deployed over Orchestrators. We emulated their milestones in terms of inputs/outputs of control events. For this task, we used servers with these characteristics: Core i5 2.0 GHz CPU, 8 GB of RAM and 64 bits Windows 7. Activities' interfaces were implemented in C#. Then, we converted them into Internet Information Service (IIS) Applications; so all the Activities' inputs were bound to SOAP-HTTP bindings and implement the notify SOAP operation. Activities' outputs triggered web actions in brokers. We developed brokers in C# and use similar bindings. Primitives publish, subscribe, un-subscribe were also implemented as SOAP operations.

The node running Trident was deployed over the Internet as well as brokers and nodes running activities. Brokers and A_L_s were grouped according to the SWf of Figure 1. For implementation purposes A_L_s under the same Local Area Network were executed by the same Orchestrator node, but they continued to use the Web interface to exchange control messages. Concerning the execution, in the SCM implementation, Trident started the execution of each one of the A_L_ using the scheduling mechanisms offered by the framework; so there was no broker participation. In the COM implementation, LG were implemented just before the successor A_L_s, and the Trident node only started the execution of A_L_-1 as well as in our solution. In the BPoPS solution we used a recursive subscription to implement the message forwarding between brokers. It was also based on SOAP-HTTP bindings. Table 2 compares the tree SCM, COM and BPoPS implementations in terms of completion time of the SWf execution, message SOAP payload, memory footprint and CPU utilization. We contemplated in all the solutions the mean, median and standard deviation, except for the message SOAP payload field, because we were using a fixed payload for this test.

The Completion time of the SWF execution and the Message SOAP payload correspond with the term Information dissemination that we introduced in Section 4.1. In this section, we described the mechanisms that allowed task gaining access to the activation of an A_L_ after a control message arrives to SWf participant. The message SOAP payload of line 2 represents the total message payload generated towards the complete execution of the SWf, including the subscription messages needed by COM and BPoPS. Trident is always taken as the reference point for all the measures. It starts the execution in AL-1 and receives the completion message from AL-11 in all the cases.

We use the Memory and CPU consumption of the SWf, gathered from the Trident, as quantitative metrics. This metrics corresponds with the Workflow monitoring characteristic mentioned in Section 4.1

From this table we extract the following conclusions:

- Regarding the Completion time of the SWf execution, BPoPS gives an average of 88,234 milliseconds, while the closer solution, the SCM, shows an average of 15,814 milliseconds. As we had different samples for this test, we applied the Mann-Whitney U test [38] for measuring the Completion time of the SWf execution. The results of this test supported the previous finding (P < 0.01). The P-values indicate that the test result is significant, so we can conclude that BPoPS offers the best performance values for this evaluation scenario.
- Concerning the Message SOAP Payload, even if BPoPS requires a higher quantity of messages, this indicator does not affect the overall SWf performance often carried out using local area networks, with low latency and stable MTU, and broadband Internet connections.
- Regarding Memory and CPU, due to the simplicity of the connection between the Trident Workflow Monitor and brokers, the BPoPS solution offers a better Memory footprint in the Monitor, an average of 104.550 MB. In terms of CPU consumption our solutions show similar values than the other solutions.

Summarizing, we analyzed the qualitative advantages of the BPoPS in comparison with the DM-based ones. In the evaluation scenario, we demonstrated that the BPoPS implementation offers similar CPU utilization than SCM and COM implementations and ≈10% improvement in the memory footprint. Finally, the test also determined that the BPoPS improves by ≈41% the completion time of the SWf execution.

## 5. Related Works

The problem of distributed execution of workflows is an open topic today. Some works [3] highlight the importance of SWfMS coordination models, not only by their nature (orchestration, choreography or mixed models) but also by the task distribution, delegation algorithms and optimizations over inter-task communication. Other works [39] compare existing SWf systems, extract the differences between the data and control planes and highly the importance of the control structures and their execution. These control structures are realized by workflow patterns.

Related to the nature of the workflow, a centralized SWfMS is often not the best solution for executing workflows as large amounts of data are routed through a centralized point which makes the workflow difficult to scale. Some approaches propose decentralized SWfMS which optimize communication by placing each orchestration engine as close as possible to the component service it manages [18], so, our work targets these kinds of systems.

For an efficient and scalable distribution of tasks some researches state that a SWf should be divided into different planes, so the participants of the workflow have to deal with different communication methods for data and control. Concerning optimization of distributed workflows, some authors [40] propose the application of data mining techniques, by carrying out a deep analysis of temporal behavior and then extracting the best way to fragment tasks depending on availability of resources. Other works [36] resolve task distribution from the perspective of workflow scheduling by elaborating P2P communication models between participants and then finding the efficient mapping of tasks. Our work differs from these approaches as it tackles the message exchange in runtime, rather than the fragmentation process while participants remain decoupled from each other. A hybrid approach for scheduling of workflows is proposed in [41], by deploying two phases. The first phase is based on clustering and grouping tasks according data links between tasks. The second phase applies a list-based heuristic to fit the allocation of task groups, according to the available resources. This approach reduces the inter-task communication costs and therefore improves the performance of the workflow execution. Nevertheless, our work focuses on how to decouple the control messages depending on the workflow relationships between parties, as we assume that data messages will be delivered using other mechanisms.

Most of the research marks grid environments as suitable scenarios for SWf execution [5], and addresses the importance of intermediate communication elements, which in our case are the brokers. Works such as [42] review the importance of the control plane of workflows and their realization using abstract languages. On this topic workflow patterns have been used [5] as the source for modeling and monitoring of workflows. Our works goes further and applies the same patterns to enhance the communication between tasks by not only monitoring, but also delivering control events.

Failures in activities can create deadlocks in workflows, so dependence between activities requires recovery methods in WfMS. In this context of Web Services recovery [43], Issarny *et al.* proposed the concept of Web Service Composition Action, which allows multiple choices to be selected based on pre-established specifications. Other works such as [44] define transaction behaviors to compensate for failures based on the workflow skeleton. In our work we also defined recovering mechanisms based on the fact that brokers manage task relationships; so it could be possible to enhance our brokers with these mechanisms. Therefore, depending on the expected runtime conditions, the amount of participants and their communication needs of the SWf, these methods could be plugged-in into our brokers.

Concerning the communication model, some works [33] develop web-based mechanisms adapted to SWfs, however, as we presented in previous sections, the communication layer can be improved by a close relationship between messaging and tasks bindings. Balis, *et al.* [10] propose a taxonomy for monitoring events, based on structured identifiers similar to our topic hierarchy. These events are managed over a Distributed Hash Table infrastructure. We consider our solution complementary [45] with this kind of infrastructure as our broker enhancements are independent from the underlying network.

The Publish/Subscribe paradigm has been applied to many scenarios such as scientific workflow interoperability [46] and recently in M2M communications [28]. Regarding the event dissemination, Alqaoud *et al.* [47] demonstrated that a topic-based Pub/Sub model, together with Web-based protocols, improves the SWf interoperability while maintains the expected loosely coupled characteristic required by highly distributed tasks. Our model shares similar characteristics and enhances this interoperability by delegating the complexity of the patterns instantiations to brokers rather than nodes running tasks.

## 6. Conclusions and Future Works

In this paper, we have addressed the problem of disseminating control events in the context of scientific workflows. For this purpose, we have proposed a model that uses workflow patterns' foundations and defined the key elements that are needed for the execution of SWf. Our model is complemented with a task interoperability reference model that allows the hierarchical organization of tasks' inputs/output while maintaining the simplicity and portability of a topic-based Pub/Sub language. We have also presented a WSDL definition that allows the configuration of brokers' interfaces, task bindings and the event layer that supports the SWf execution. A reference broker model is also provided as well as procedures brokers can carry out in order to recover relationships between tasks, inside the SWf.

The proposed model is qualitatively analyzed and compared with current SWf solutions in Section 4, where advantages of the new model are stated. Thus, existing implementation are categorized according to their communication mechanisms and interactions among tasks and schedulers. The qualitative areas were evaluated from the workflow pattern perspective, their triggering conditions and control events generated and disseminated. Therefore, we showed that our model offer the strongest qualitative advantages in terms of information dissemination, tasks decoupling and filtering of control events. We performed an evaluation of a realistic SWf scenario using the Trident Workflow Workbench and demonstrated that our model offered better quantitative advantages in terms of completion time of the SWf execution, maintained a lower memory footprint and similar CPU utilization.

In future research we will be working on workflow fragmentation issues and mechanisms for supporting patterns that head to directed cyclic graphs. We will work on brokers capable of supporting patterns by employing auto-installable and auto-configurable libraries. Finally, we will also investigate the characterization of workflow internal structures and their optimal instantiation and execution using adapted gossip algorithms and protocols.

## Figures and Tables

**Table 1.** SWf execution requirements and communication solutions.

	**Semi-Centralized MesSaging (SCM)**	**Distributed Messaging ExChange (DM)**	**Pub/Sub with Channel Optimization Messaging (COM)**	**Bounded Patterns over Pub/Sub (BPoPS)**
**Information dissemination**	*Sync messaging Async Messaging*	*Request-response Async Messaging*	*Partial Asynchronous*	*Fully Asynchronous*
**Information dispatching and filtering**	*Application Level*	*Application Level*	*Event level*	*Event Level*
**Tasks decoupling through workflow patterns**	*Partial*	*Partial*	*Partial*	*Complete*
**Flexibility over workflow changes**	*Depending on activity-to-activity links*	*Depending on Single links*	*Depending on number of activities*	*Workflow dependent*
**Scalability of events**	*Depending on number of activities*	*Node dependent*	*Pattern dependent*	*Topology dependent*
**Workflow monitoring and failure handling**	*Semi-centralized*	*Shared space*	*Distributed – task level*	*Distributed – broker level*

**Table 2.** Quantitative evaluation of the evaluation scenario.

	**Semi-Centralized Messaging (SCM)**			**Pub/Sub with Channel Optimization Messaging (COM)**			**Bounded Patterns over Pub/Sub (BPoPS)**		

	Mean	Median	SD	Mean	Median	SD	Mean	Median	SD
**(1) Completion time of the SWf execution (ms)**	*15,181*	*10,537*	*202*	*15,182*	*10,538*	*202*	*8,823*	*8778*	*162*
**(2) Message SOAP payload (bytes)**	*8,041*	-	-	*16,510*	-	-	*21982*	-	-
**(3) Memory footprint (MB)**	*117.124*	*117.718*	*3.234*	*119.582*	*119.652*	*1.3976*	*104.550*	*104.773*	*2.740*
**(4) CPU utilization (%)**	*15.069*	*14.739*	*3.941*	*14.993*	*14.965*	*5.729*	*15.538*	*16.102*	*3.978*
